# Acoustoelectric Effect on the Responses of SAW Sensors Coated with Electrospun ZnO Nanostructured Thin Film

**DOI:** 10.3390/s120912006

**Published:** 2012-08-30

**Authors:** Cihat Tasaltin, Mehmet Ali Ebeoglu, Zafer Ziya Ozturk

**Affiliations:** 1 Materials Institute, TUBITAK Marmara Research Center, Gebze, Kocaeli 41470, Turkey; E-Mail: cihat.tasaltin@tubitak.gov.tr; 2 Department of Electrical & Electronic Engineering, Dumlupinar University, Kutahya 43100, Turkey; E-Mail: mebeoglu@dumlupinar.edu.tr; 3 Department of Physics, Gebze Institute of Technology, Gebze, Kocaeli 41470, Turkey; E-Mail: zozturk@gyte.edu.tr

**Keywords:** surface acoustic waves, zinc oxide, nanostructures, electrospun, acoustoelectric effect

## Abstract

In this study, zinc oxide (ZnO) was a very good candidate for improving the sensitivity of gas sensor technology. The preparation of an electrospun ZnO nanostructured thin film on a 433 MHz Rayleigh wave based Surface Acoustic Wave (SAW) sensor and the investigation of the acoustoelectric effect on the responses of the SAW sensor are reported. We prepared an electrospun ZnO nanostructured thin film on the SAW devices by using an electrospray technique. To investigate the dependency of the sensor response on the structure and the number of the ZnO nanoparticles, SAW sensors were prepared with different coating loads. The coating frequency shifts were adjusted to fall between 100 kHz and 2.4 MHz. The sensor measurements were performed against VOCs such as acetone, trichloroethylene, chloroform, ethanol, *n*-propanol and methanol vapor. The sensor responses of *n*-propanol have opposite characteristics to the other VOCs, and we attributed these characteristics to the elastic effect/acoustoelectric effect.

## 1. Introduction

To date, many research groups have studied metal oxide (such as ZnO, SnO_2_, TiO_2_, In_2_O_3_, WO_3_) thin film coated SAW gas sensors to detect CO, H_2_S, HCHO, NH_3_, H_2_, ethanol, methanol and RH [1–4]. Recently, metal oxide nanostructures (such as nanoparticles, nanotubes, nanowires, nanobelts) have attracted attention for chemical sensors as they have unique surface, small-size, and quantum effects. Metal oxide nanostructures typically have a faster response and a lower limit of gas detection due to their very high surface-to-volume ratios. Zinc oxide is a particularly interesting material for semiconductors due to its wide band gap (3.37 eV) and has been studied in many areas such as catalysts, electronics, optoelectronics and gas sensing material.

There are many theoretical and practical studies on SAW sensors. The gas sensing mechanism of a SAW sensor can be explained as follows: when the sensitive layer absorbs ambient gas molecules, the boundary conditions for the propagating surface are changed, and consequently, both the velocity and attenuation of the wave change. These changes can be detected with great accuracy by a SAW oscillator as a frequency shift. The propagation velocity of the surface wave depends on various factors. The sensor responses are frequency shifts due to a mass loading. This mass loading depends on the volume of the sensitive material V_f_ and the concentration of the solution of the sensitive material C_s_. The partition coefficient K represents the ratio between C_s_ and the concentration of the vapor C_v_. The mass loading Δm can be calculated as:

(1) $$\Delta \text{m}={\text{C}}_{\text{s}}{\text{V}}_{\text{f}}=\text{K}{\text{C}}_{\text{v}}{\text{V}}_{\text{f}}$$

For a SAW oscillator, the frequency shift Δf due to a mass loading is given as follows:

(2) $$\Delta \text{f}=({\text{k}}_{1}+{\text{k}}_{2}){\text{f}}_{0}^{2}\frac{\Delta \text{m}}{\text{A}}=({\text{k}}_{1}+{\text{k}}_{2}){\text{f}}_{0}^{2}\text{hK}{\text{C}}_{\text{v}}$$

where k_1_ and k_2_ are constants of the piezoelectric substrate, *h* is the coated sensitive film thickness, f_0_ is the unperturbed resonant frequency of the SAW oscillator and A is the sensitive film area [5,6].

The mass, conductivity and elasticity of the sensing materials are three key parameters in the sensing mechanism of a SAW. In the case of a gas, the sensitive material is a thin film; the propagation of the waves is much perturbed due to the adsorption of gas molecules at the surface of the film. At this point, the main effects that perturb the SAW propagation can be given as the mechanical effects, the electrical effects and the atmospheric effects. As mentioned above, the mechanical effects include the mass, thickness, coefficients of elasticity and coefficient of viscosity of the sensitive film. The electrical effects of the sensitive film are the conductivity, permittivity, mobility, diffusion constant of the charge carrier in the sensitive film and concentration of the majority carriers [7,8]. Furthermore, the atmospheric effects are the temperature, pressure and humidity. From the practical point of the view, only the following two effects have potential meaning for a gas-sensitive film: a change in the mass density of the film and a change in its electrical conductivity.

The acoustoelectric effect of a SAW sensor has been studied by many researchers. Since the early investigations, there has been a wealth of research on the SAW acoustoelectric effect on metals and metal oxides. Ricco *et al.* studied the relationship between the conductive sensing film and SAW responses in 1985 [6]. Fisher *et al.* gave an explanation for the relationship between the film thickness and the acoustoelectric effect. According to Fisher, thin films can be classified into three regimes, such as ultra-thin, thin, and thick films. These regimes are primarily distinguished by the electrical conduction mechanisms and elasticity of the sensitive film. Some films are composed of a discontinuous network of atomic clusters in which the primary method of electrical conduction is thermally activated quantum tunneling across the discontinuities [9]. Jakubik and Penza gave significant reports on this classification [4,10,11]. Jakubik reported that, for SAW sensors, the region around the highest wave attenuation region is more sensitive. This clustering explanation is similar to Fisher's explanation. In addition, film conductivity is one of the parameters of wave attenuations. Therefore, the acoustoelectric effect can be seen in the highest wave attenuation region, which is the highest possible.

The total effect of a relative change of the wave propagation (Δv/v_0_) and attenuation (Δα/k) can be given as:

(3) $$\frac{\Delta \alpha }{\text{k}}=\frac{{\text{K}}^{2}}{2}\frac{{\text{v}}_{0}{\text{C}}_{\text{s}}\sigma_{\text{s}}}{\sigma_{\text{s}}^{2}+{\left({\text{v}}_{0}{\text{C}}_{\text{s}}\right)}^{2}}+4{\text{C}}_{\text{e}}\frac{{\text{f}}_{0}}{{\text{v}}_{0}^{2}}(\Delta \text{h}{\text{G}}^{\prime \prime })$$

(4) $$\frac{\Delta \text{v}}{{\text{v}}_{0}}=-{\text{C}}_{\text{m}}{\text{f}}_{0}\rho_{\text{s}}+4{\text{C}}_{\text{e}}\frac{{\text{f}}_{0}}{{\text{v}}_{0}^{2}}(\Delta \text{h}{\text{G}}^{\prime })-\frac{{\text{K}}^{2}}{2}\frac{\sigma_{\text{s}}^{2}}{\sigma_{\text{s}}^{2}+{\left({\text{v}}_{0}{\text{C}}_{\text{s}}\right)}^{2}}$$

where G″ is loss module, G′ is storage module, K^2^ is the electromechanical effect, σ_s_ = σh is surface conductivity, σ is bulk conductivity, and C_s_ = ε_p_ + ε_o_ is the total dielectric permittivity of the substrate and the environment. The interdependence between the electrical potential associated with the SAW and the carriers of the electric charge in the gas-sensitive film leads to a decrease in the velocity. This effect depends on the electromechanical effect K^2^ and the surface conductivity of the layer σ_s_ but is independent of the frequency of the wave propagation f_o_. The changes of the wave attenuation have a resonant characteristic [5].

In this study, the acoustoelectric effect on the responses of SAW sensors that are coated with electrospun ZnO nanostructured films was investigated with acetone, trichloroethylene, chloroform, ethanol, propanol and methanol.

## 2. Experimental

### 2.1. Sensor Preparation

For the sensor coating, 0.01 M Zn(CH_3_COO)_2_·2H_2_O was dissolved in methanol by stirring for 2 h. The solution was then coated on the 433 MHz frequency dual-port resonator Rayleigh-SAW devices (SAW Components GmbH, Dresden, Germany) mounted on a TO-39 socket using an electrospray technique, and the coated film annealed at 500 °C in the air atmosphere using conventional furnace for 2 h to obtain the zinc oxide form.

**Figure 1.**
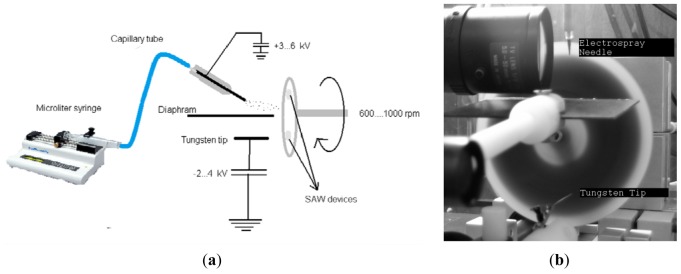
(**a**) Schematic diagram of electrospraying system. (**b**) Photograph of the electrospraying system.

The electrospraying technique is a two-compartment setup with a sample holder that rotates at a velocity of 1,000 rpm, exposing the sensors to the positive electrospray mist and a negative discharge cloud. The coating voltages were adjusted to ∼+3.5 kV for the needle and ∼−1.5 kV for the tungsten tip. The coating voltage values were adjusted to maintain a stable Taylor cone. A schematic diagram and a photograph of the electrospraying system is given in Figure 1. Using this method, soluble materials can be coated on nonconductive surfaces [12,13]. The spray was not applied directly to the sample holder but at a 45° angle. In this method, only small charged droplets hit the sensor surface, whereas larger droplets do not reach it due to their larger inertia. A microsyringe pump (KD Scientific Inc., Holliston, MA, USA) was used to provide a continuous flow of the coating solution. The coating frequency shifts were adjusted in the range of 100 kHz–2.4 MHz. The upper limit of the frequency shift was determined by the wave propagations transmission between IDTs. When the mass loading was increased slightly due to the coating, the wave propagation stopped.

### 2.2. Gas Sensor Measurement Setup

The gas stream containing gas vapor was generated from cooled bubblers that were immersed in a thermally controlled bath with synthetic air as the carrier gas. The gas stream, which was saturated with the analyte, was then diluted with pure synthetic air to adjust the gas concentration to the desired amount by using computer driven mass flow controllers (MKS Instruments Inc, Andover, MA, USA) at a constant flow rate of 200 mL/min. Sensors were tested by isothermal gas exposure experiments at 22 °C. Typical experiments consisted of repeated exposure to the analyte gas (20 min) and a subsequent purging with pure air (20 min) to reset the baseline. During the measurements, the gas concentration was increased in the presence or absence of humidity. The SAW sensor system is homemade and capable of testing 6 sensors. The sensors are housed in temperature-controlled chambers that are 4 mL in volume. For the SAW sensor array, the frequencies of the individual sensors are read out sequentially using a multiplexing technique. A seventh, uncoated SAW is used as a reference which was closed with a lid to block the influence of the gases.

## 3. Results and Discussion

### 3.1. Structural Characterization of Electrospun ZnO Nanostructured Film

**Figure 2.**
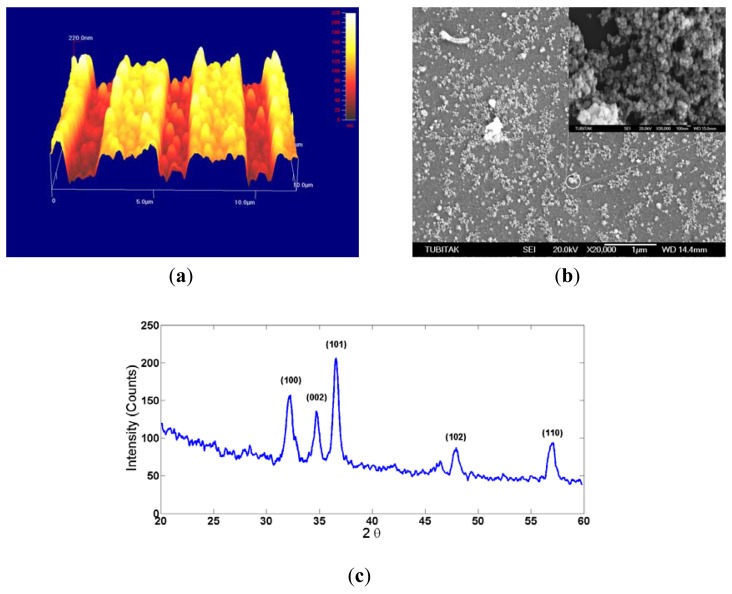
(**a**) AFM image. (**b**) SEM image. (**c**) XRD spectrum of the electrospun ZnO thin film.

The morphology and crystallinity of the electrospun ZnO nanostructured thin film was analyzed by AFM, SEM and XRD (Figure 2). The AFM image was taken from the region on IDT transducers; Figure 2(a) shows that the nanoparticles were not fully filled between the fingers of the IDT, and some discontinuous network clusters were shown. The SEM image was taken from the region between the two IDTs (Figure 2(b)) and shows that the ZnO nanoparticles were approximately 50 nm in diameter. The nanoparticles were not located in a strict pattern in the conductance path, so we believe that electron transportation between the fingers of the IDT cannot be achieved. Therefore, the coated sensitive film has a low conductivity. To obtain the XRD spectrum of the electrospun ZnO thin film, we prepared a thicker film on the quartz substrate (Figure 2(c)). The peaks were indexed as polycrystalline ZnO with a hexagonal wurtzite structure [14]. No peaks from any other phase of ZnO or from impurities were observed.

### 3.2. Characterization of the ZnO-Coated SAW Sensor

**Figure 3.**
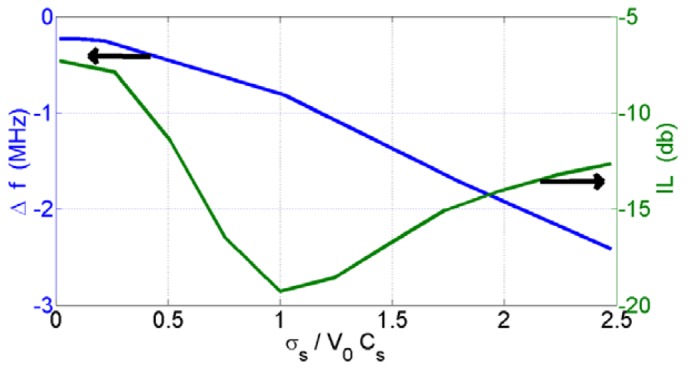
The conductivity graph *versus* the insertion loss (IL) and coating thickness.

The conductivity of the sensing film is one of the parameters that change the frequency shift of the SAW device. It depends on how the nanoparticle clusters formed on the SAW. Therefore, the structure of the sensing film has a key effect on the characterization of the SAW sensor. To investigate the sensor response and the SAW device's characterization dependency on the metal oxide nanoparticle structure formed on the sensor surfaces, we prepared SAW sensors with different coating loads of the metal oxides. The conductivity graph versus the insertion loss (IL) and coating thickness are shown in Figure 3. It was shown that a bend around the highest value of attenuation was observed. The highest attenuation of the prepared SAWs was found for $\frac{\sigma_{\text{s}}}{{\text{v}}_{0}{\text{c}}_{\text{s}}}=1$, and σ_s=_v_0_c_s_ = 0.9 × 10^−6^ Ω^−1^ was calculated according to the conductivity and the scattering parameter measurements. The active range of the acoustoelectric effect of the SAWs (the highest change of wave velocity) was found in the range of $0.4<\frac{\sigma_{\text{s}}}{{\text{v}}_{0}{\text{C}}_{\text{s}}}<1.7$. This range also includes the maximum point of insertion loss (IL). These results are similar to those of Jakubik *et al.* [15,16].

The SAW sensor structure with different coating films can create new possibilities for detecting different gas molecules with the same materials as the acoustoelectric effect on the SAW sensor structure changes according to the film conductivity and elasticity. Consequently, we can use an acoustoelectric effect between the surface wave and the SAW sensor structure with different coating films. This effect can be many times greater than the mass effect, which is dominant in non-conductive polymer films, simple metal and dielectric films in SAW-based gas sensors [17–19].

### 3.3. Sensor Measurements

Sensor measurements were performed against VOCs such as acetone, trichloroethylene, chloroform, ethanol, *n*-propanol and methanol vapor. The concentrations of each gas were varied in the range of 100–5,000 ppm (Table 1). The sensor responses increased linearly with an increasing analyte concentration. For a better comparison, the responses of different vapor pressures' relative concentrations p_i_/p_0i_ are used, where p_i_ is the actual analyte concentration and p_0i_ is the saturation vapor pressure at the measurement temperature [20].

#### 3.3.1. Sensor Responses

**Figure 4.**
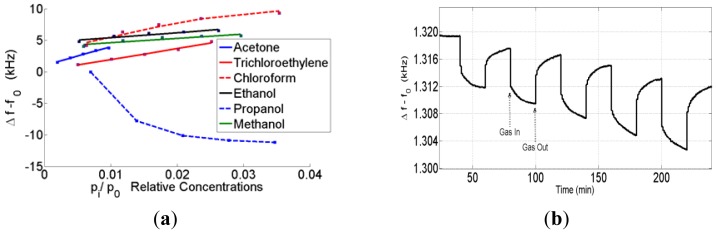
The sensor responses against (**a**) VOCs (**b**) propanol in the active region.

The characteristics of the sensors changed depending on the acoustoelectric effect. Some sensors that are in this range have an opposite response to some analytes such as propanol. Therefore, the SAW sensor response depends on three parameters: the mass, the conductivity and the elasticity of the sensing film. According to Fisher's explanation, the electrical conduction mechanisms and elasticity properties of the film depend on how the nanoparticle clusters formed on the SAW. The ZnO nanoparticles on the SAW have a discontinuous structure at low frequency shifts. Therefore, the electrical conductivity of the film was lower than the expected values. By increasing the coating loads, the discontinuity of the nanoclusters can decrease, and a few conductivity paths can be created. Consequently, the elasticity part of the equation $4{\text{C}}_{\text{e}}\frac{{\text{f}}_{0}}{{\text{v}}_{0}^{2}}(\Delta \text{h}{\text{G}}^{\prime })$ can play a dominant role in the frequency shift in the active region, which is the reason for the opposite sensor responses. The sensor responses against the VOCs in the active region are shown in Figure 4(a,b).

**Figure 5.**
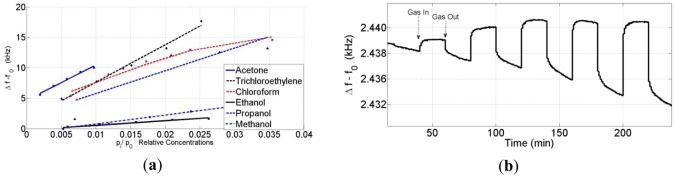
The sensor response profiles against (**a**) VOCs (**b**) propanol that have the highest coating shifts.

The sensor responses against the VOCs and propanol in the active region are shown in Figure 4. In the case of metal oxides, the conductivity cannot play a dominant role as seen by the fact that, when the conductivity is changing against VOCs, the sensor needs to heat to 200∼300 °C. By increasing the coating loads, the mass effect becomes the dominant effect on the sensor response profile. The sensor response profiles against the VOCs and propanol that have the highest coating shifts are shown in Figure 5.

#### 3.3.2. Humidity Influence on Sensor Responses

**Figure 6.**
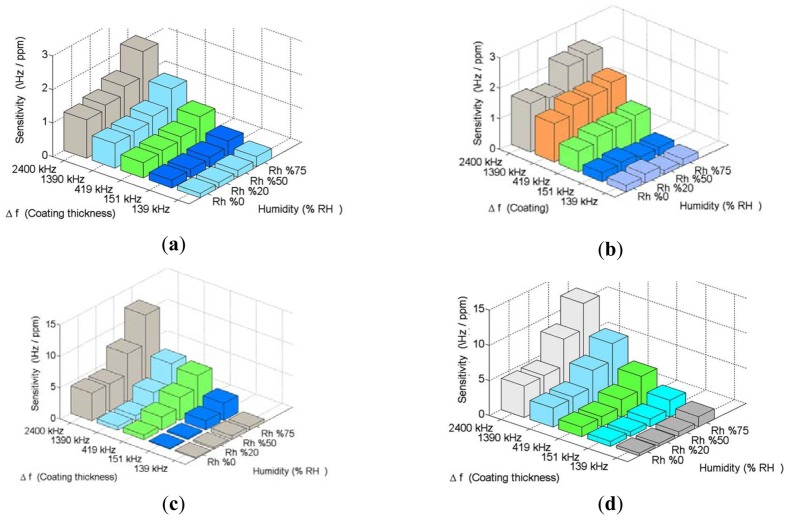
The sensitivities of the VOCs at the different humidity levels for (**a**) Acetone (**b**) Chloroform (**c**) Propanol (**d**) Trichloroethylene.

To further investigate the influence of humidity on the SAW devices, the sensors were exposed to VOCs at humidity levels from 20 to 75% rh. The sensor responses were also investigated in repeated measurements. An increase of the sensitivity has been observed for acetone, chloroform, propanol and trichloroethylene with an increased humidity level of the carrier gas. By increasing the humidity, the methanol and ethanol responses decreased. The sensitivity was changed depending on the coating frequency shift and the highest variations in the active region. The sensitivities of the VOCs at the different humidity levels are shown in Figure 6(a–d). The bar graph shows the sensitivities to the measured gases at different levels of background humidity.

### 3.4. Constituting of Sensor Array and Data Evaluations

**Figure 7.**
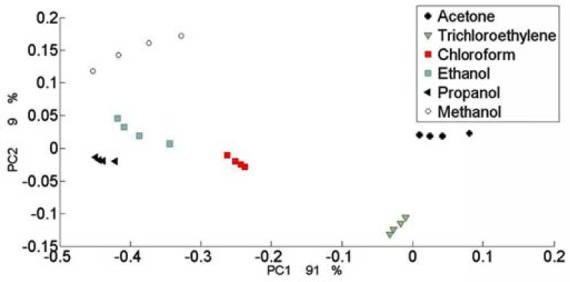
A biplot of loadings and scores in the PCA.

The similarity of the sensors was explored using principal component analysis (PCA). This method allows the similarities or dissimilarities among the individual sensors to be explored according to their responses. The goal of the evaluation is to identify the possible variations in the sensor characteristics caused by the changes of nanoparticle structures on the SAW. To obtain the best PCA classification, each sensor must have a different sensing profile compared to others. Thus, five different coated SAWs were selected as a sample of all the sensors that we had. The PCA was performed with the help of MATLAB and the sensor response after normalization and centering. A biplot of the loadings and scores in the PCA is shown in Figure 7. The first two principal components contain approximately 100% of the total variance.

The analytes were found to be separated on the PCA mapping. The alcohols were located a far distance from the other compounds and closer to each other. Acetone and trichloroethylene were located opposite to the alcohols. On the other hand, each analyte group was located in a different area on the PCA map.

## 4. Conclusions

The above described results show that an account of the mass and elastic loadings is generally necessary. The different contributions can combine subtractively or additively depending on the elastic parameter of the film, their variations due to a gas absorption of the SAW device. In particular for a given film material and gas, it become possible to increase, decrease or cancel the SAW sensor response.

The preparation of an electrospun ZnO nanostructured thin film on a SAW sensor and the investigation of the acoustoelectric effect on the SAW sensor are described. ZnO is a very good candidate for improving the sensitivity of gas sensor technology. Chloroform, propanol, acetone, and the humidity detection performance of the nanostructured ZnO thin film were investigated in detail. SAW sensors with metamorphosed structures of sensing coating that were formed by the same material generate different sensor responses. These results could help create a sensor array prepared using the same sensing material but having different sensor profiles.

## Figures and Tables

**Table 1.** Properties of analytes: saturation vapor pressure at −10 °C, the tested concentration range, the saturation vapor pressure at the measurement temperature of 22 °C as calculated using Antoine's equation.

**Analyte**	**Concentration (ppm)**		**p^0^(−10 °C)**	**p^0^(22 °C)**
	**Min.**	**Max.**	**ppm**	**ppm**
**Acetone**	1,360	6,800	54,400	270,000
**Trichloroethylene**	550	3,100	15,800	87,000
**Chloroform**	1,150	5,800	46,200	220,000
**Ethanol**	460	2,300	9,200	66,200
**Propanol**	132	660	2,650	22,300
**Methanol**	515	4,120	20,600	140,000
